# Acute hemorrhagic demyelination in a murine model of multiple sclerosis

**DOI:** 10.1186/1742-2094-5-31

**Published:** 2008-07-07

**Authors:** Istvan Pirko, Georgette L Suidan, Moses Rodriguez, Aaron J Johnson

**Affiliations:** 1 Department of Neurology and Neuroscience, University of Cincinnati, Cincinnati, Ohio, USA; 2 Department of Neurology and Immunology, Mayo Clinic, Rochester, Minnesota, USA

## Abstract

Acute hemorrhagic leukoencephalomyelitis (AHLE) is a rare neurological condition characterized by the development of acute hemorrhagic demyelination and high mortality. The pathomechanism of AHLE, as well as potential therapeutic approaches, have remained elusive due to the lack of suitable animal models. We report the first murine model of AHLE using a variation of the Theiler's Murine Encephalitis Virus (TMEV) MS model. During acute TMEV infection, C57BL/6 mice do not normally undergo demyelination. However, when 7 day TMEV infected C57BL/6 mice are intravenously administered the immunodominant CD8 T cell peptide, VP2_121–130_, animals develop characteristics of human AHLE based on pathologic, MRI and clinical features including microhemorrhages, increased blood-brain barrier permeability, and demyelination. The animals also develop severe disability as assessed using the rotarod assay. This study demonstrates the development of hemorrhagic demyelination in TMEV infected C57BL/6 mice within 24 hours of inducing this condition through intravenous administration of CD8 T cell restricted peptide. This study is also the first demonstration of rapid demyelination in a TMEV resistant non-demyelinating strain without transgenic alterations or pharmacologically induced immunosuppression.

## Findings

The acute monophasic demyelinating disorders, including acute disseminated encephalomyelitis (ADEM) and acute hemorrhagic leukoencephalitis (AHLE) usually present 1–3 weeks after infections or vaccination, but have also been observed without preceding illness [1,2]. In cases of ADEM, the prognosis is favorable, with 60–80% of cases experiencing complete recovery [3,4]. However, AHLE is associated with rapidly deteriorating focal and diffuse neurological symptoms leading to death within 2–14 days [5-8]. The few patients that survive AHLE usually have significant residual neurological symptoms [9]. Its high mortality and poor response to therapy necessitate the development of animal models of AHLE to understand the mechanism of its pathology.

During acute TMEV infection of the H-2^b ^haplotype, 50–70% of central nervous system (CNS) infiltrating CD8^+ ^T cells have T cell receptor specificity towards an immunodominant viral peptide VP2_121–130 _presented in the context of the D^b ^class I molecule [10]. We have previously reported that a rapidly fatal hemorrhagic CNS disease develops in the C57BL/6 strain when the immunodominant VP2_121–130 _peptide is intravenously administered 7 days post TMEV infection [11]. In these studies, we confirmed by northern blot analysis that TMEV RNA in the CNS was not increased in animals administered VP2_121–130 _peptide, demonstrating that this fatal condition was not due to increased viral load [11]. The C57BL/6 strain is considered non-demyelinating, as these mice do not develop chronic viral persistence and demyelination [12]. We now report that the fatal hemorrhagic CNS disease in these mice is associated with demyelination. Our findings highlight two very important concepts: 1) a classically non-demyelinating strain can develop fulminant hemorrhagic demyelination by intravenous administration of an immunodominant peptide recognized by CD8 T cells; and 2) this hyperacute model of hemorrhagic demyelination is the first TMEV-induced murine model of AHLE.

All experiments were approved by the Institutional Animal Care and Use Committee of the University of Cincinnati. All adequate measures were taken to minimize pain or discomfort, and experiments were conducted in accordance with international standards on animal welfare as well as being compliant with local and national regulations. TMEV infection was induced via intracranial injection of 2 × 10^6 ^PFUs of TMEV [11]. Induction of the hemorrhagic demyelinating condition required IV injection of 0.1 mg VP2_121–130 _peptide 7 days after infection. D^b ^binding Human papilloma virus E7 peptide was used as negative control [11].

**Figure 1:**
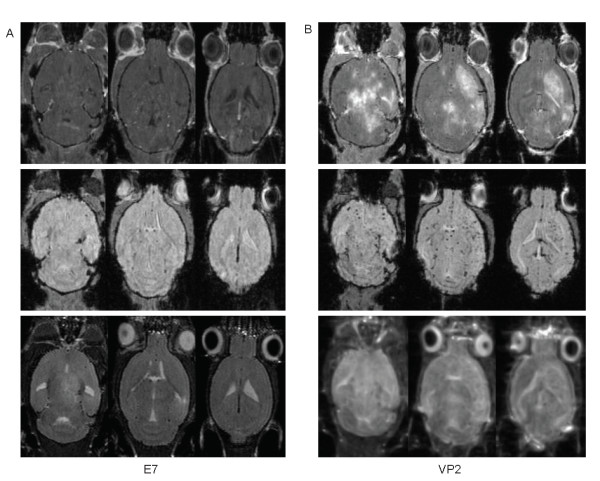
In vivo MRI images of 8 day TMEV infected C57BL/6 mice, 24 hours after VP2_121–130 _peptide injection (right panel, B) or irrelevant E7 peptide injection (left panel, A). Top row: axial images extracted from the gadolinium enhanced T1 weighted dataset demonstrate extensive contrast enhancement of confluent areas of the brain in the VP2 injected mouse, and very faint enhancement in the parahippocampal area in the E7 injected animal. Middle row: T2* weighted images demonstrate punctuate T2 hypointensities, corresponding with areas of microhemorrhages in the VP2 injected mouse. Bottom row: T2 weighted images demonstrate T2 hyperintensities, corresponding with areas of edema, inflammatory infiltrates, demyelination and tissue damage in the VP2 injected mouse; minimal hyperintense changes are also demonstrated in the parahippocampal areas of the E7 injected animal.

(1) In vivo magnetic resonance imaging was performed in a 7 Tesla narrow bore small animal imaging system (Bruker Biospin, Billerica, MA). Inhalational isofluran anesthesia was used. A custom made saddle coil was used for acquisition and excitation [11,13]. We acquired three dimensional T2 weighted (RARE pulse sequence, TR1500 ms, TE65 ms, FOV: 4 × 2.5 × 2.5 cm, matrix: 256 × 128 × 128, RARE factor 16), T2* weighted (GEFI pulse sequence, TR150 ms, TE10 ms, FA:15, FOV: 4 × 2.5 × 2.5 cm, matrix: 256 × 128 × 128, NEX 4) and T1 weighted (SE pulse sequence, TR200 ms, TE10 ms, FOV: 4 × 2.5 × 2.5 cm, matrix: 256 × 128 × 128, NEX 2) images. Gadopentetate dimeglumine (Magnevist, Bayer Healthcare Pharmaceuticals) was injected intravenously 20 minutes before acquiring the post-contrast images, using a human equivalent dose of 0.1 mmol/kg. Analysis of the images, including 2D slice extraction was done using Analyze 8.0 [14]. Figure 1 shows MRI images obtained 24 hours after VP2 peptide injection in 7 day TMEV infected C57BL/6 mice (B). The MRI images revealed extensive areas of hyperintense signal abnormality on T2 weighted images, corresponding with edema, cell infiltration and demyelination in the deep white matter, the corpus callosum, and in gray matter structures. The T2* weighted images revealed several areas of punctuate microhemorrhages in the areas where T2 hyperintensities were also demonstrated. The T1 weighted post-gadolinium images demonstrate significant blood-brain barrier permeability in large confluent areas of the brain. MRI images obtained 24 hours after E7 peptide injection (A) revealed very subtle T2 hyperintensities and minimal contrast enhancement in the parahippocampal and deep gray matter areas, where the majority of viral replication and the resulting immune response occurs. No T2* hypointensities are demonstrated in E7 peptide injected animals, suggesting that microhemorrhages are not part of the pathology in sham treated animals. Also at 24 hours, mice were assessed using the rotarod apparatus, a rotating bar which increases from 5–40 RPM over 7 minutes. The rotarod is a well documented method of determining sensorimotor deficits and overall functional impairment in mice [15,16]. VP2 peptide treated animals demonstrated greatly decreased ability to negotiate the rotarod apparatus as compared to E7 peptide treated controls (P < .001, data not shown).

(2) To determine the presence and extent of demyelination, mice were perfused via intracardiac puncture with 50 mL Trump's fixative solution. Brains were removed and post-fixed for an additional 24 hours in Trump's fixative. Coronal blocks of brain tissue were osmicated and embedded in glycol methacrylate [17]. Two-μm-thick sections were stained with a modified paraphenolyene diamine stain to detect demyelination.

**Figure 2:**
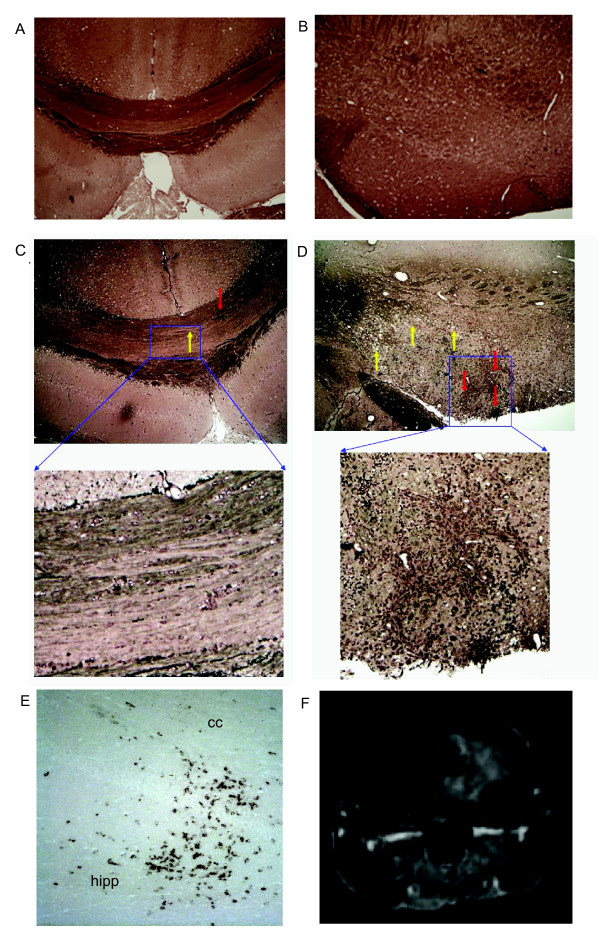
Pathology of mouse brain coronal sections of 7 day TMEV infected C57BL/6J mice. A and B: 24 hours post intravenous irrelevant E7 peptide injection. C and D: 24 hours post intravenous VP2_121–130 _peptide injection. Red arrows demonstrate microhemorrhages, yellow arrows demonstrate sites of demyelination. E: CD8 T cell stain in the corpus callosum (cc) and hippocampus (hipp) 24 hours post intravenous VP2_121–130 _peptide injection. F: T1 Gadolinium enhanced MRI at the level of the hippocampus 24 hours post intravenous VP2_121–130 _peptide injection.

We used pathologic studies of animals administered mock E7 peptide or VP2 peptide treatment to determine if these signal changes were indicative of demyelination. 24 hours after E7 or VP2 peptide injection in 7 day TMEV infected C57BL/6 mice, brains were harvested and processed for histology using paraphenolyene diamine stain for demyelination. Figure 2 shows extensive demyelination in areas adjacent to microhemorrhages in VP2 treated animals (C, D). Demyelination was observed in both the corpus callosum as well as in the deep white matter. Additional demyelination was observed in and adjacent to gray matter structures. No such abnormalities were observed in mock E7 peptide treated controls (A, B). To assess the presence of CD8 T cells in areas with demyelination and gadolinium enhancement we used immunoperoxidase staining for CD8a (Ly-2) as previously described [18]. In Figure 2 we show that CD8 T cells were found throughout the brain, most notably in the hippocampal regions and corpus callosum (E). Gadolinium-enhanced T1 weighted MRI showed evidence of BBB disruption in these same areas of the brain (F). These experiments demonstrated the presence of CD8 T cells in the corpus callosum, a region with high levels of demyelination and BBB permeability.

We report that immunodominant peptide injection in TMEV infected C57BL/6 mice causes significant blood brain barrier (BBB) permeability and CNS damage, resulting not only in inflammatory infiltrates, microhemorrhages, and tissue damage, but also demyelination. To our knowledge, this is the first example of inducing demyelination in a non-demyelinating mouse strain in the TMEV model without the use of immunosuppressants or transgenic technology [19]. Our previously published results also demonstrate that CD8+ T cells specific for the immunodominant D^b^: VP2 _121–130 _epitope play a key role in mediating the observed pathology [11].

Demyelination is rapid in this murine model, occurring within 24 hours of intravenous injection of VP2 peptide. Also of interest, demyelination, as a result of TMEV infection, is normally not observed in the C57BL/6 strain. Susceptibility to demyelination in the TMEV model is largely dependent on the major histocompatibility class I genotype of the mouse [12]. However, other quantitative trait loci (QTL) pertaining to susceptibility to persistent virus infection and chronic demyelination in the spinal cord have also been defined [12]. C57BL/6 mice, being of the resistant H-2^b ^haplotype, mount a very strong antiviral response, mediated by epitope specific CD8+ T-cells recognizing the VP2_121–130 _viral capsid fragment [20]. This results in clearance of the virus, and survival of the animal without neurological deficits. As demonstrated by this study, a demyelinating syndrome can be rapidly induced by a simple in vivo injection of the immunodominant peptide VP2 which is recognized by the majority of brain infiltrating CD8 T cells [10].

In the human condition, ADEM is characterized by multifocal perivascular demyelination in an asymmetric fashion, mostly in the white matter. However, gray matter involvement of the basal ganglia, thalamus and brainstem has also been reported. In AHLE, necrotizing immune infiltration, perivascular demyelination, ball and ring hemorrhages, prominent infiltrates with lymphocytes, macrophages and neutrophils have been reported [2]. Radiologically AHLE is also more severe than ADEM, with mass effect, edema, and punctuate hemorrhages being present adjacent to usually asymmetric T2 hyperintense lesions. The histological and MRI findings put forth in this study clearly present with these traits, demonstrating that in vivo activation of CNS infiltrating CD8 T cells can serve as a novel model of AHLE. Such studies may ultimately lead to the development of more effective and focused therapies instead of non-specific and partially effective use of steroids [21], cyclophosphamide or plasma exchange [22]. Future experiments directed at putative mechanisms of BBB disruption and demyelination are already underway in our labs.

## Competing interests

The authors declare that they have no competing interests.

## Authors' contributions

IP was responsible for conception, experimental design, MRI, interpretation of results and for writing the manuscript. GS isolated tissue, performed CD8 T cell stain, performed behavioral assays (rotarod), assisted with preparation of manuscript. MR was responsible for demyelination study and preparation of manuscript. AJ was responsible for experimental design, preparation of manuscript, infection of animals, administration of peptide.
